# Acupuncture for chronic tinnitus: study protocol for a clinical trial with auditory brainstem response outcomes

**DOI:** 10.3389/fneur.2026.1928456

**Published:** 2026-09-10

**Authors:** Shiyu Zhang, Minghui Zhao, Kaili Chen, Shenglu Xu, Keqin Ye, Lianqiang Fang, Hong Gao

**Affiliations:** 1 The Third Clinical Medical College, Zhejiang Chinese Medical University, Hangzhou City, China; 2 Department of Otorhinolaryngology, the Third Affiliated Hospital of Zhejiang Chinese Medical University, Hangzhou City, China; 3 Department of Acupuncture and Moxibustion, the Third Affiliated Hospital of Zhejiang Chinese Medical University, Hangzhou City, China

**Keywords:** ABR (auditory brainstem response), acupuncture, clinical trial, protocol, tinnitus

## Abstract

**Background:**

Chronic tinnitus is a common clinical condition with no universally accepted effective treatment option. Increasing evidence suggests that dysfunction within the auditory pathway may contribute to tinnitus pathophysiology, and auditory brainstem response (ABR) has been proposed as a potential objective measure of auditory neurophysiological function. Acupuncture is widely used for tinnitus management; however, the relationship between acupuncture-associated symptom changes and objective electrophysiological alterations remains unclear.

**Objective:**

This study aims to investigate differences in ABR parameters between patients with chronic tinnitus and healthy controls and to explore whether clinical changes following acupuncture intervention are accompanied by alterations in auditory neurophysiological measures.

**Methods:**

This study is designed as a prospective two-part hybrid study protocol consisting of a matched case–control observational component and a longitudinal self-controlled intervention component. A total of 36 patients with bilateral chronic tinnitus and 36 age- and sex-matched healthy controls will be recruited. Both groups of participants will undergo auditory brainstem response (ABR) assessments at baseline and after treatment timepoint; in order to assess test–retest variability, the healthy control group will undergo two ABR measurements. Patients with tinnitus will receive 10 acupuncture treatments over a 4-week period. Clinical outcome measures, including the Tinnitus Handicap Inventory (THI), the Tinnitus Impact Questionnaire (TEQ), and the Tinnitus-Related Visual Analog Scale, will be assessed at baseline, after treatment, and at the 8-week follow-up.

**Expected results:**

This study will characterize baseline differences in ABR parameters between individuals with chronic tinnitus and healthy controls and will examine whether acupuncture-associated improvements in tinnitus symptoms are accompanied by changes in auditory neurophysiological measures. The findings will provide preliminary data regarding the feasibility of ABR as an objective outcome measure for future tinnitus intervention studies.

**Conclusion:**

This protocol describes an exploratory clinical study designed to evaluate clinical and neurophysiological changes associated with acupuncture intervention in chronic tinnitus. The results may contribute to a better understanding of objective electrophysiological assessment in tinnitus research and provide preliminary evidence to support future controlled clinical trials.

**Trial registration:**

https://clinicaltrials.gov/, identifier [NCT07522567]

## 1 Introduction

Tinnitus is described as the subjective perception of sound in the absence of an external auditory stimulus. It is frequently characterized as a hissing, buzzing, or ringing sound within the head or ears and can generally be divided into two categories: subjective and objective. Subjective tinnitus, which accounts for the vast majority of cases, is perceived only by the patient and cannot be heard by an examiner. Objective tinnitus, in contrast, is rare and can be auscultated by an examiner, typically arising from somatosensory or vascular causes. Tinnitus can also be categorized as primary (idiopathic, without an identifiable underlying cause) or secondary (associated with a specific identifiable pathology, such as otologic, neurologic, or metabolic disorders) (1). It is a widespread issue that is quite prevalent around the world. According to a 2016 comprehensive systematic review, the reported prevalence of tinnitus varies considerably depending on its definition, ranging from 5.1% to 42.7% globally (2). While transient tinnitus is common, a substantial proportion of individuals experience persistent symptoms. In mainland China, the incidence of tinnitus varied from 4.3% to 51.33%, which was in line with data from around the world. The incidence of tinnitus rose with age, yet no statistically significant sex difference was observed (3). Insomnia, anxiety, depression, and a lower quality of life (QoL) are associated with the severity of tinnitus (4, 5). Furthermore, tinnitus is linked to depression, sleeplessness, cognitive impairment and reduced speech comprehension (6), even in individuals with clinically normal hearing; in fact, 43.8% of tinnitus patients have mental comorbidities (7). Despite its high prevalence and profound impact (8), the underlying pathophysiological mechanisms of tinnitus remain incompletely understood, and no universally effective pharmacological or interventional cures are currently available.

According to the widely adopted clinical classification (9), tinnitus is categorized by duration as acute (<3 months), subacute (3–6 months), and chronic (>6 months). Emerging evidence suggests that chronic tinnitus is not merely a consequence of peripheral cochlear dysfunction but is closely linked to maladaptive neuroplasticity within the central auditory system (10). The prevailing pathophysiological model proposes that tinnitus results from diminished cochlea sensory input, which triggers compensatory homeostatic gain changes along the auditory pathway (11, 12). Moreover, the emotional distress and psychiatric comorbidities commonly associated with tinnitus are increasingly recognized as arising from altered connectivity between auditory and limbic structures, including the amygdala and hippocampus (13). Specifically, even in those with normal audiometric thresholds, disruption to inner hair cell ribbon synapses can decrease afferent input to the auditory nerve, increasing central gain at the brainstem and auditory cortex levels (14, 15). Animal research provides a supporting basis for this process. These studies show that noise exposure can induce cochlear synaptic lesions, which is typically characterized by selective loss of high thresholds auditory nerve fibers. At the same time, an increased spontaneous neural activity and enhanced neural synchrony have also been observed in the thalamus and auditory cortex (16). In general, at least two distinct subtypes of tinnitus are recognized: cochlear tinnitus and central tinnitus. This distinction is supported by the observation that tinnitus is not always abolished following cochlear nerve section (17). Cochlear tinnitus is a kind of tinnitus caused by abnormal cochlear nerve activity. While central tinnitus, in contrast, originates at the cortical level within the central auditory pathways, independent of cochlear nerve activity (12).

The auditory brainstem response (ABR) has become a useful non-invasive method for evaluating the brainstem and auditory nerve pathways’ functional integrity (18). Wave I of ABR originates from the distal portion of the auditory nerve and reflects the summed activity of cochlear afferent fibers, as confirmed by direct intracranial recordings in humans (19), while wave V of ABR is mostly produced in the lateral lemniscus and inferior colliculus, signifying neural processing in the midbrain. A few publications showed that ABR abnormalities are linked to the perception of tinnitus (20). According to Bramhall et al. (21), there is a substantial correlation between a lower ABR wave I amplitude and a greater chance of reporting tinnitus in young individuals with normal audiometric thresholds and normal outer hair cell activity. This association remained after controlling for sex and DPOAE levels. This result supports the theory that the ensuing central gain may strengthen cochlear synapses, which are indicated by a drop in wave I amplitude, hence causing tinnitus (22). Recently, Edvall et al. (23) found that the prolongation of wave V latency in ABR can distinguish between individuals with persistent tinnitus and those who occasionally have tinnitus or no tinnitus. The results show that the transformation from intermittent perception to continuous awareness is related to functional reorganization at the midbrain level. Despite the above progress, there are still no objective biomarkers that can effectively monitor the changes caused by treatment. Most previous findings have been demonstrated at the group level, and the ability of ABR parameters to discriminate tinnitus status or predict individual treatment response remains uncertain. In addition, the exact relationship between electrophysiological changes and clinical symptom characteristics is still not fully clear.

The pathophysiological process of tinnitus is still poorly understood, and the lack of known core causes makes therapy very difficult. For this condition, which affects millions of people, existing treatments including a variety of interventions such as sound therapy, cognitive behavioral therapy, and medication—are effective, but there is no universally effective “gold standard.” Treatment responses vary from person to person, and patients with similar tinnitus characteristics often experience varying degrees of benefit (24). Furthermore, no drugs have been approved by the European Medicines Agency or the U.S. Food and Drug Administration (FDA) to treat tinnitus (25). While carrying out these neurophysiological studies, acupuncture has also become a promising treatment option for chronic tinnitus. As a major treatment modality of traditional Chinese medicine, acupuncture is widely used in East Asia and has gained more and more recognition around the world because of its high patient acceptance and good safety. Systematic reviews and meta-analyses have reported promising but not definitive results for acupuncture in tinnitus (26), and the quality of the existing evidence base remains relatively limited, and methodological flaws have been noted in many of the included studies (27). In order to make up for the above shortcomings, this study pursues two main goals. The first goal is to compare ABR parameters between patients with chronic tinnitus and healthy controls who match their age and sex, so as to explore the relationship between the function of auditory conduction pathways and chronic tinnitus. The second is to measure the ABR parameters and the scores of TEQ (Tinnitus Effects Questionnaire), THI (Tinnitus Handicap Inventory) scales and the Tinnitus-Related Visual Analog Scale. Measurements will be taken before and after 10 acupuncture treatments, as well as at the 8-week follow-up. This allows us to evaluate the therapeutic effect of acupuncture on patients with chronic tinnitus, and explore the possible neurophysiological processes behind it. Notably, emerging preclinical evidence suggests that acupuncture may exert modulatory effects on auditory function at multiple levels of the auditory pathway. For instance, electroacupuncture at the Zhongzhu acupoint (TE3) has been shown to promote neuroplasticity of the central auditory pathway, including the brainstem and auditory cortex, as evidenced by significant changes in auditory brainstem response parameters in both animal models and human studies. These findings provide a neurophysiological rationale for investigating whether acupuncture can modulate ABR parameters in human tinnitus patients (28). Therefore, we propose the following specific hypotheses: Hypothesis 1 (cross-sectional): Patients with chronic tinnitus will show abnormal characteristics on ABR compared with age- and sex-matched healthy controls, reflecting aberrant auditory pathway function. Hypothesis 2 (longitudinal): Following 10 sessions of acupuncture, patients with chronic tinnitus will show significant improvements in THI scores (primary clinical outcome) and concomitant changes in ABR parameters (primary neurophysiological outcome), particularly wave V latency. Hypothesis 3 (association): Changes in ABR parameters from baseline to post-treatment will correlate with changes in clinical outcome measures (THI, TEQ, VAS), supporting a neurophysiological basis for acupuncture’s effects on tinnitus.

## 2 Method

### 2.1 Design overview

This study adopts a hybrid two-part design comprising a matched case–control observational component and a self-controlled interventional component.

In the first component, patients with chronic tinnitus will be compared with age- and sex-matched healthy controls to identify baseline differences in auditory neurophysiological characteristics, including ABR parameters. In the second component, patients with chronic tinnitus will receive a standardized acupuncture intervention and will be followed longitudinally to evaluate changes in subjective tinnitus severity and auditory neurophysiological function. Healthy controls will also undergo two ABR assessment over a comparable time interval to estimate test–retest variability and distinguish treatment-associated changes from normal measurement variability.

There is no sham acupuncture control group in this study. We acknowledge that placebo effects, expectation-related responses, and other non-specific effects associated with acupuncture cannot be completely excluded and may influence the interpretation of treatment-associated changes. To minimize the placebo effect, a strict assessor-blinded design will be adopted. The ABR waveforms will be anonymized and coded before interpretation, with audiologists blinded to both group allocation and time points. Clinical scale interviewers will be blinded to time point, and data analysts will work on a fully coded dataset until the final analysis is locked.

**Figure 1:**
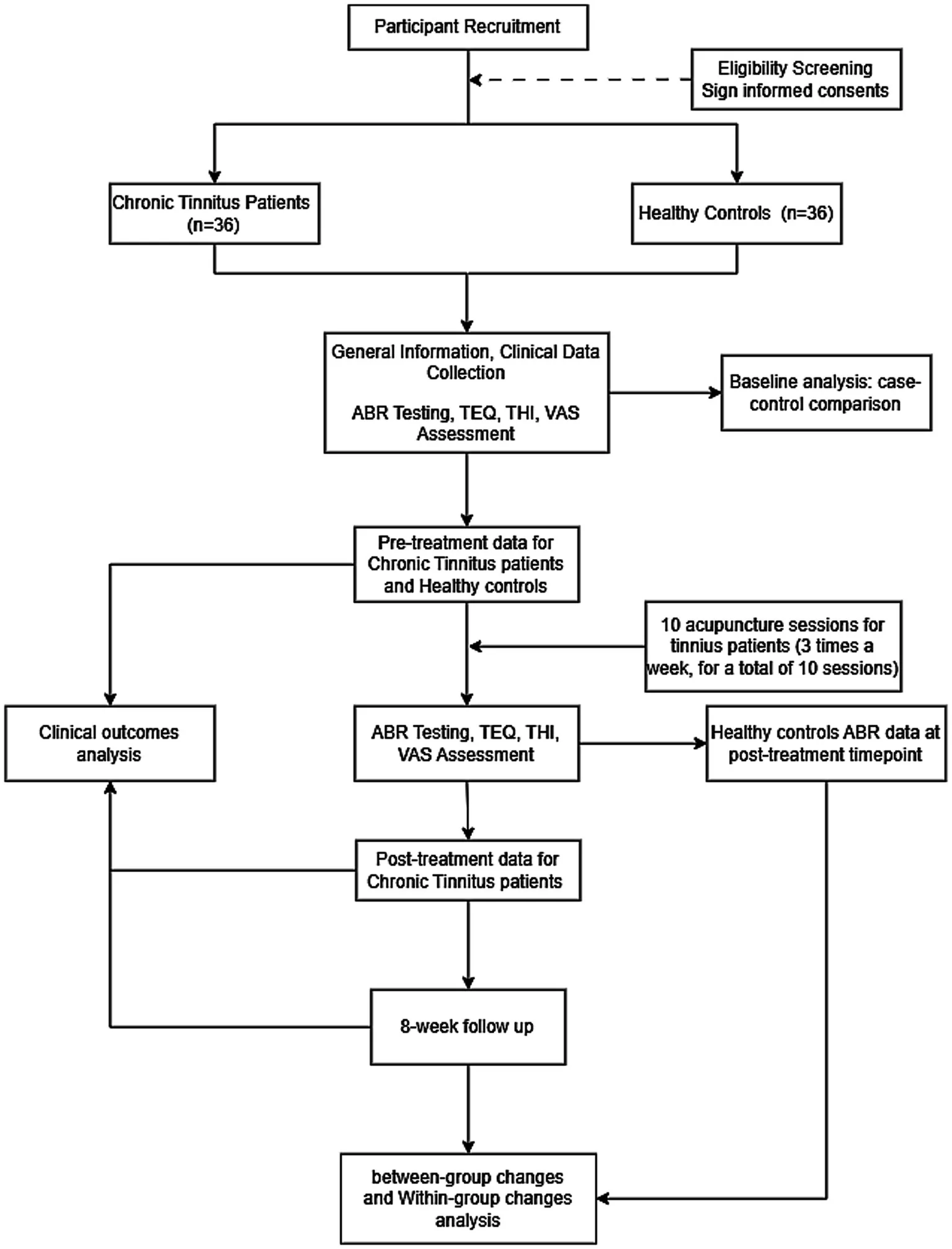
Flow chart of the study process.

In addition, although the following measures will not reduce the placebo effect, we will take further steps to ensure the overall scientific rigor and interpretability of the study results. Baseline characteristics of all participants will be compared to verify the comparability between the tinnitus and healthy control groups. During recruitment, participants will be provided with standardized information regarding the study procedures, potential benefits, possible adverse reactions, and the sensory experiences associated with acupuncture. The information will be presented in a balanced manner to ensure adequate understanding of the intervention without emphasizing potential treatment outcomes. Furthermore, subjective questionnaire data will be evaluated alongside objective ABR measures to provide complementary information regarding clinical changes and associated neurophysiological alterations. The Standard Protocol Items: Recommendations for Interventional Trials (SPIRIT) guidelines will be followed in the development of the protocol (29). Zhejiang Chinese Medical University’s Third Affiliated Hospital will host the study. A total of 72 participants will be included in this study, including 36 patients with chronic tinnitus and 36 healthy controls who matched the patient in age and sex. Healthy controls will be individually matched with tinnitus participants according to sex and age within ±3 years. In addition to demographic matching, we will also compare PTA and audiogram characteristics between the two groups at baseline, as well as before and after treatment within each group. If there are significant intergroup differences in audiogram characteristics, these specific frequencies will be included as covariates in the statistical model used to compare auditory brainstem response (ABR) parameters between groups. The treatment plan includes 10 sessions of acupuncture, which can be completed within 4 weeks and carried out three times a week (usually 2 days apart between each treatment). There are three time points used to evaluate tinnitus-related clinical outcomes and auditory brainstem response (ABR) parameters, namely the baseline period (before the start of treatment), the end of the course (within 1 week after the tenth treatment), and the 8th week after the completion of treatment as follow-up point. The Institutional Ethical Review Board (IRB) of the Third Affiliated Hospital of Zhejiang Chinese Medical University has granted ethical permission for the trial (ZSLL-KY-2025-032-01), which will be conducted in accordance with the Declaration of Helsinki. The Ethics Committee will be notified right away of any amendments to the study design. NCT07522567 is the identification code for this trial’s registration in the Clinical Trials Registry. Table 1 displays the study’s SPIRIT list, while Figure 1 displays the flow diagram.

**Table 1:** SPIRIT schedule of enrollment, interventions, and assessments.

	Study period							
	Enrolment	Allocation	Treatment period				Post-treatment	Follow-up
TIMEPOINT (week)	Week −1(T0)	Week 0	W1	W2	W3	W4	W5 (T1)	W13 (T2: 8-week FU)
Enrolment								
Eligibility screen	X							
Informed consent	X							
Demographic/medical history	X							
Allocation		X						
Interventions								
Acupuncture (10 sessions, 3×/week)			X	X	X	X		
Assessments								
ABR parameters	X						X	
Pure-Tone Threshold testing	X							
Tympanometry & Acoustic reflex testing	X							
DPOAEs	X							
Otoscopic examination	X							
THI	X						X	X
TEQ	X						X	X
Loudness/Annoyance VAS	X						X	X
Adverse events			X	X	X	X	X	

### 2.2 Subjects recruitment

Patients with chronic tinnitus as well as healthy volunteers will be recruited from the Third Affiliated Hospital of Zhejiang Chinese Medical University’s departments of neurology, otolaryngology, and acupuncture and moxibustion.

### 2.3 Inclusion and exclusion criteria of tinnitus patients

#### 2.3.1 Inclusion criteria

1) Diagnosis of bilateral chronic subjective tinnitus (including primary and secondary tinnitus, as well as cochlear and central cases), with a duration of at least 6 months, and a score of at least 16 on the Chinese version of the THI scale (mild) (30);
2) Pure-tone average hearing threshold (PTA) < 20 dB HL in both ears, with no individual frequency exceeding 30 dB HL (31);
3) Normal tympanometric findings, acoustic reflection test findings, sotoscopic examination findings and normal Distortion product otoacoustic emissions (DPOAEs);
4) Aged between 18 and 60 years, inclusive, any sex;
5) Both tympanic membranes are intact and the external auditory canal is clear;
6) Sufficient expressive and comprehensive language skills, adequate cognitive function, and the capacity to collaborate with the study’s experimental procedures;
7) Signing the informed consent form and not taking part in any other interventional clinical trials at this time.

#### 2.3.2 Exclusion criteria

1) Drug addiction, alcoholism, and mental illnesses (including comprehension difficulties, full aphasia, and severe cognitive disorders);
2) Serious systemic illnesses, such as cardiovascular or cerebrovascular disease, or severe abnormalities of the central nervous system; these conditions will be identified through the participant’s self-report during the screening interview and, upon the participant’s consent to access existing medical records, confirmed by medical records or previous diagnostic imaging studies.
3) A history of craniocerebral trauma or other documented organic neurological abnormalities;
4) Abnormal tympanometric findings, acoustic reflection test findings, otoscopic examination findings or abnormal DPOAEs;
5) Ménière’s disease, otosclerosis, acoustic neuroma, abrupt sensorineural hearing loss, conductive and mixed hearing loss, or other conditions affecting the inner ear or auditory nerve;
6) History of objective tinnitus, somatosensory tinnitus, external ear canal infection, acute or chronic otitis media, or other conditions affecting the outer or middle ear;
7) Participants who are currently receiving or have recently received other tinnitus-related interventions, including pharmacological treatment, sound therapy, psychological interventions, or other acupuncture treatments;
8) Unable to tolerate acupuncture treatments due to various reasons, as well as pregnant and lactating women.

### 2.4 Inclusion and exclusion criteria of healthy volunteers

#### 2.4.1 Inclusion criteria

1) Pure-tone average hearing threshold <20 dB HL in both ears, with no individual frequency exceeding 30 dB HL;
2) Normal tympanometric findings, acoustic reflection test findings, otoscopic examination findings and normal DPOAEs.
3) Aged between 18 and 60 years, inclusive, any sex;
4) Both tympanic membranes are intact and the external auditory canal is clear;
5) Sufficient expressive and comprehensive language skills, adequate cognitive function, and the capacity to collaborate with the study’s experimental procedures;
6) Signing the informed consent form and not taking part in any other interventional clinical trials at this time.

#### 2.4.2 Exclusion criteria

1) Pregnant or breastfeeding women, and those who have a request for childbearing within the next 6 months;
2) History of head trauma, or history of persistent tinnitus (defined as tinnitus lasting >5 min, occurring more than once per week, or lasting >3 months);
3) Abnormal tympanometric findings, acoustic reflection test findings, otoscopic examination findings or abnormal DPOAEs;
4) Any condition that precludes ABR testing, such as the existence of metallic implants in the body;
5) Participation in any ongoing interventional clinical trials.

If a person satisfies all inclusion requirements and none of the exclusion requirements, they will be qualified to take part in the study. Written informed consent will be obtained from all participants prior to enrollment. Participants will be informed of the study purpose, procedures, potential risks and benefits, and their right to withdraw at any time without consequence.

### 2.5 Audiological assessments

Pure-tone audiometry will be performed using an AC40 clinical audiometer (Interacoustics A/S, Middelfart, Denmark) with TDH-39 supra-aural headphones and a B-71 bone conductor, in a sound-attenuated booth complying with ISO 8253-1 standards. Air conduction thresholds will be obtained at 0.125, 0.25, 0.5, 1, 2, 4, and 8 kHz, and bone conduction thresholds at 0.25, 0.5, 1, 2, and 4 kHz, using the modified Hughson-Westlake procedure. The PTA will be calculated as the average of thresholds at 0.5, 1, 2, and 4 kHz. Individual frequency thresholds and the configuration of the audiogram (i.e., flat, sloping, high-frequency dip, or notch at 4000 Hz) will be recorded and described for both groups.

Tympanometry and acoustic reflex testing will be performed using an Interacoustics Titan IMP440 impedance system (Interacoustics A/S, Middelfart, Denmark). Tympanometry will be conducted using a 226 Hz probe tone to assess middle ear compliance and pressure. Acoustic reflex thresholds will be measured ipsilaterally and contralaterally at 500, 1,000, 2,000 and 4,000 Hz.

Distortion product otoacoustic emissions (DPOAEs) will be recorded using an Interacoustics Titan DPOAE440 system (Interacoustics A/S, Middelfart, Denmark). DPOAEs will be measured to assess outer hair cell function, and will be recorded across a frequency range of 1–8 kHz (1/3 octave steps) using primary tone levels of L1 = 65 dB SPL and L2 = 55 dB SPL (f2/f1 ratio = 1.22). A DPOAE response will be considered present if the signal-to-noise ratio (SNR) is ≥6 dB at the corresponding frequency. DPOAEs will be considered normal if responses are present at ≥6 of the 8 tested frequencies (1, 1.5, 2, 3, 4, 5, 6, and 8 kHz). Notably, absent DPOAEs at high frequencies (≥6 kHz) may occur in asymptomatic individuals with normal pure-tone thresholds and do not necessarily indicate pathology; such findings will be documented but will not automatically exclude participants unless accompanied by other abnormalities.

Tinnitus Handicap Inventory (THI) will be administered using the validated Chinese (Mandarin) version (32).

### 2.6 Intervention

#### 2.6.1 Treatment course

All chronic tinnitus patients will receive a total of 10 acupuncture sessions, administered once every 2 days (three times per week) over a period of 4 weeks. Based on our prior research and clinical experience using acupuncture to treat tinnitus, we selected a total of 10 acupuncture sessions (33, 34). These methods are thought to be representative and appropriate for assessing acupuncture’s effectiveness in treating persistent tinnitus.

Patients will be observed at week eight after completing 10 sessions in order to more fully assess the long-term efficacy of acupuncture treatment.

#### 2.6.2 Acupuncture program

##### 2.6.2.1 Acupuncture points prescription

Based on the clinical expertise of senior acupuncturists and the results of earlier relevant investigations, the acupoint prescription for this study was created (35). The selected acupoints are as follow: Yifeng (bilateral), Tinggong (bilateral), Tinghui (bilateral), Zhongwan, Xiawan, Qihai, Guanyuan, Yindu (bilateral), Shangqu (bilateral), Huaroumen (bilateral). Licensed acupuncturists with over 10 years of clinical experience will deliver all treatments. Before the experiment, all practitioners will receive standardised instructions to ensure protocol adherence. A brochure outlining the standardized operating procedures will be provided to each practitioner. Table 2 summarizes the precise positions of each acupoint, which will be identified in accordance with the WHO Standard Acupuncture Point Locations (36).

**Table 2:** Acupoint positions.

Acupoints	Position
Yifeng (TE17)	In the anterior region of the neck, posterior to the ear lobe, in the depression anterior to the inferior end of the mastoid process.
Tinggong (SI19)	On the face, in the depression between the anterior border of the centre of the tragus and the posterior border of the condylar process of the mandible.
Tinghui (GB2)	On the face, in the depression between the intertragic notch and the condylar process of the mandible.
Zhongwan (CV12)	On the upper abdomen, 4 B-cun superior to the centre of the umbilicus, on the anterior median line.
Xiawan (CV10)	On the upper abdomen, 2 B-cun superior to the centre of the umbilicus, on the anterior median line.
Qihai (CV6)	On the lower abdomen, 1.5 B-cun inferior to the centre of the umbilicus, on the anterior median line.
Guanyuan (CV4)	On the lower abdomen, 3 B-cun inferior to the centre of the umbilicus, on the anterior median line.
Yindu (KI19)	On the upper abdomen, 4 B-cun superior to the centre of the umbilicus, 0.5 B-cun lateral to the anterior median line.
Shangqu (KI17)	On the upper abdomen, 2 B-cun superior to the centre of the umbilicus, 0.5 B-cun lateral to the anterior median line.
Huaroumen (ST24)	On the upper abdomen, 1 B-cun superior to the centre of the umbilicus, 2 B-cun lateral to the anterior median line.

##### 2.6.2.2 Acupuncture procedures

The patients will be placed in a supine position. Acupuncture needles (Hwato brand, Suzhou Medical Devices, China) measuring 50 mm in length and 0.30 mm in diameter will be utilized for the three periauricular acupuncture points (Tinggong, Tinghui, and Yifeng) following a standard disinfection of the surrounding skin. The needles for Tinggong and Tinghui will be inserted to a depth of about 20 to 25 mm, vertical to the skin. The needle tip for Yifeng will be inserted at a 45° angle and pointed anterosuperiorly in the direction of the ear canal. The needle will be gently inserted to a depth of approximately 20–25 mm, followed by manipulation with small-amplitude, low-frequency twisting (amplitude 180°, frequency <60 times/min) to elicit the De Qi sensation. When the patient reports a distinct sensation of soreness, numbness, or distension radiating into the ear canal, the manipulation is continued. Acupuncture needles (40 mm in length, 0.25 mm in diameter) will be used for limb acupoints, with lifting-thrusting and even twisting techniques. The Chinese Modified Massachusetts General Hospital Needle Sensation Scale (C-MASS) will be used to measure the degree of needle sensation. Each needle is stored for 30 minutes.

#### 2.6.3 ABR data collection

The Interacoustics Eclipse EP25 auditory evoked potential system (Interacoustics A/S, Middelfart, Denmark) with click acoustic stimulation will be utilized for recording auditory brainstem responses. Every recording will take place in a calm, sound-attenuated, electrically insulated space that is kept at a suitable temperature. At the beginning of the experiment, subjects will be asked to lie in a supine position in a comfortable state and to remain relaxed or fall asleep if possible. The ambient noise level in the sound-attenuated room will be maintained at ≤30 dB(A). The skin should be cleansed with a scrub before electrode application. The ground electrode is located at the low forehead, the reference electrodes on either side of the mastoid process, and the recording electrode at midline of the forehead close to the hairline. Electrode impedance should not exceed 3 kΩ.

*Stimulus and recording parameters*: The stimulus will be a 0.1 ms square-wave click with alternating polarity (rarefaction and condensation) to minimize stimulus artifact. The stimulus repetition rate will be 11.1 clicks per second (Hz), chosen because it produces stable and reproducible ABR waveforms with minimal neural adaptation effects, and it is a standard default rate widely used in clinical and research settings (37). The maximum stimulus intensity will be 100 dB nHL. Bandpass filter settings: Low-pass: 1,500 Hz, High-pass: 33 Hz, 6/oct. Artifact rejection: Sweeps containing activity exceeding ±40 μV will be automatically rejected. Analysis time window: A 10 ms post-stimulus epoch will be recorded. Number of sweeps: A minimum of 1024 artifact-free sweeps will be averaged for each waveform, with duplicate recordings obtained to ensure reproducibility.

*Stimulus ear*: For patients with bilateral tinnitus, ABR will be recorded separately for each ear using monaural click stimulation with contralateral white noise masking (at 40 dB below the stimulus level) to prevent crossover responses. The order of ear testing will be randomized across participants.

*Analytical strategies*: Two complementary analytical approaches will be employed. First, ABR thresholds will be determined by descending intensity series starting from 100 dB nHL in 10 dB decrements, with the threshold defined as the lowest stimulus intensity at which a reproducible wave V can be visually identified. Second, supra-threshold ABR waveform parameters will be analyzed at a fixed intensity of 100 dB nHL, including peak latencies (waves I, III, and V), wave amplitudes, and inter-peak latencies (I–III, III–V, and I–V).

A minimum of two stable waveforms with good reproducibility will be obtained at maximum intensity and at threshold. All waveforms and data will be independently interpreted and analyzed by two licensed audiologists who will remain blinded to group allocation.

### 2.7 Outcome measures

The outcomes of this study were designed to evaluate both the clinical efficacy and neurophysiological changes associated with acupuncture treatment in patients with chronic tinnitus. Considering the different characteristics of subjective symptom assessment and electrophysiological evaluation, clinical and neurophysiological outcomes will be analyzed separately.

The assessment schedule is summarized in Table 1. Patient-reported outcomes, including tinnitus severity and related functional impairment, will be assessed at baseline (T0), after completion of acupuncture treatment (T1), and at the 8-week (after the beginning of acupuncture treatment) follow-up visit (T2). Auditory brainstem response (ABR) measurements will be performed in both the healthy control group and the tinnitus patient group at baseline (T0) and after treatment completion (T1), as the primary purpose of the electrophysiological assessment is to evaluate changes in auditory pathway function during the course of treatment. ABR testing will not be conducted at the follow-up visit, as the primary purpose of the follow-up assessment is to evaluate the durability of subjective tinnitus improvement.

#### 2.7.1 Primary outcomes

##### 2.7.1.1 Primary clinical outcome

The primary clinical outcome will be the change in the Tinnitus Handicap Inventory (THI) score from baseline to post-treatment and follow-up.

The THI is a quick, simple, and psychometrically sound tool that assesses how tinnitus affects day-to-day functioning. The THI has 25 questions with three main subscales (affective, functional, and severity scores) and a total scale score of 0–100, with lower scores indicating less severe tinnitus-related handicap. It has been shown to be an accurate and significant indicator of self-reported tinnitus-related discomfort (38).

##### 2.7.1.2 Primary neurophysiological outcome

The primary neurophysiological outcome will be the change in ABR wave V latency from baseline to post-treatment.

Previous studies have suggested that alterations in ABR latency parameters may represent objective neurophysiological changes associated with tinnitus-related auditory dysfunction. Wave V latency was chosen because it exhibits the highest test–retest reliability among ABR parameters and is the most clinically robust and widely reported metric in human auditory brainstem assessment. Although wave I amplitude is mechanistically important and is discussed in the introduction as a candidate biomarker of cochlear synaptopathy, its variability is substantially influenced by individual anatomical factors, making it less suitable as a primary endpoint for sample size estimation.

#### 2.7.2 Secondary outcomes

##### 2.7.2.1 Secondary clinical outcomes

1) Tinnitus effects questionnaire: One of the most used instruments for evaluating tinnitus is the Tinnitus Effects Questionnaire (TEQ). The six factors that comprise the composite score used to measure the severity of the tinnitus are the environment in which it occurs, its duration, its effect on sleep and work-study, its disruption of mood, and the patient’s overall assessment of the tinnitus’s severity. More severe tinnitus is indicated by higher scores. The scale is broken down into five grades: Grade I (1–6 points), Grade II (7–10 points), Grade III (11–14 points), Grade IV (15–18 points), and Grade V (19–21 points) (39). The TEQ will be used as a complementary tinnitus-specific questionnaire to evaluate tinnitus characteristics and symptom burden. Compared with THI, which primarily focuses on functional and emotional consequences, TEQ provides additional information regarding tinnitus perception and daily-life impact. Its shorter completion time and applicability in Chinese clinical populations make it suitable as a supplementary patient-reported outcome measure.
2) Tinnitus loudness and annoyance visual analogue scales (VAS): Two 0–10 VAS scores will be collected to evaluate subjective tinnitus loudness and tinnitus-related annoyance. Higher scores indicate greater perceived loudness or distress. These simple and sensitive measures will provide additional information regarding short-term symptom changes that may not be fully captured by standardized questionnaires (40).

##### 2.7.2.2 Secondary neurophysiological outcomes

Secondary neurophysiological outcomes will include additional ABR parameters, including: wave I amplitude and latency, wave III amplitude and latency, wave V amplitude, interpeak latencies (I–III, III–V, and I–V).

### 2.8 Statistical analysis

Statistical analyses will be performed using SPSS version 27.0 (IBM Corp., Armonk, NY, USA). All statistical tests will be two-sided, with a significance level of *p* < 0.05. Baseline demographic and clinical characteristics will be summarized using descriptive statistics. Continuous variables will be presented as mean ± standard deviation (SD) or median (interquartile range, IQR), depending on data distribution, while categorical variables will be expressed as frequencies and percentages. Normality of continuous variables will be assessed using the Shapiro–Wilk test and visual inspection of Q–Q plots. For the control group, ABR measurements will be performed at two time points (T0 and T1, matching the interval of the patient group) to assess test–retest variability. This allows us to distinguish treatment-related changes from normal variability. For bilateral ABR measurements, the primary analysis will be conducted at the participant level by averaging measurements from both ears.

For baseline comparisons between tinnitus participants and healthy controls, paired-sample t-tests or Wilcoxon signed-rank tests will be used according to data distribution, because controls will be individually matched with tinnitus participants based on age and sex.

Longitudinal changes in ABR parameters will be analyzed using two-way mixed-design analysis of variance (ANOVA), with group (chronic tinnitus vs. healthy control) as a between-subject factor and time (baseline vs. post-treatment/retest assessment) as a within-subject factor. If significant between-group differences in audiometric profiles are detected at baseline or within each group, corresponding frequency-specific hearing thresholds will be included as covariates in the analyses. The primary parameter of interest will be the group × time interaction effect, which will determine whether changes in ABR parameters over time differ between tinnitus participants and healthy controls. If the interaction is significant, simple main effects will be examined with Bonferroni correction. Intraclass correlation coefficients (ICC, two-way random-effects model) will be calculated for each ABR parameter in healthy controls to assess test–retest reliability. Within-group changes between baseline and post-treatment/retest assessment will also be examined using paired-sample *t*-tests or Wilcoxon signed-rank tests when appropriate.

Clinical outcomes, including THI, TEQ, and tinnitus-related VAS scores, will be evaluated only in tinnitus participants using one-way repeated-measures ANOVA across baseline, post-treatment, and 8-week follow-up. When assumptions of normality or sphericity are violated, appropriate non-parametric tests or Greenhouse–Geisser corrections will be applied. Associations between changes in ABR parameters and changes in subjective tinnitus severity will be explored using Spearman rank correlation analysis. For secondary analyses involving multiple ABR parameters, false discovery rate (FDR) correction using the Benjamini-Hochberg procedure will be applied to control for multiple comparisons.

Missing data will be assessed and reported. Linear mixed-effects models or multiple imputation methods will be considered for sensitivity analyses if substantial missing longitudinal data occur.

### 2.9 Sample size estimation

The two integrated components of this mixed-design study underwent separate sample size estimations. Calculations were conducted using PASS software (version 16.0, NCSS, Kaysville, Utah, USA), and a 20% attrition rate was accounted for in both calculations.

For the matched case–control observational component, the sample size was estimated based on expected differences in ABR wave V latency, which was selected as the primary electrophysiological endpoint for this comparison. Based on preliminary observations from an exploratory pilot assessment, a mean paired difference in wave V latency of 0.08 ms with a standard deviation of the differences of 0.14 ms was assumed. The preliminary assessment was primarily designed to evaluate the feasibility of ABR measurement and estimate potential differences in ABR parameters between tinnitus participants and healthy controls. Using a paired-sample t-test with a two-sided α level of 0.05 and a statistical power of 80%, a minimum of 30 matched pairs (60 total participants) is required. Accounting for a 20% attrition rate, 36 pairs (72 total participants: 36 patients and 36 matched healthy controls) will be recruited.

For the self-controlled interventional component (within the patient group), the sample size was calculated based on anticipated changes in the Tinnitus Handicap Inventory (THI) score, which serves as the primary clinical endpoint for evaluating treatment efficacy. Based on our pilot study and previous clinical observations (35), a mean reduction in THI score of 12.07 points with a standard deviation of 23.28 points after treatment is anticipated. We acknowledge that this standard deviation is relatively large compared to the estimated mean change, which reflects the substantial clinical heterogeneity inherent in chronic tinnitus patients with respect to baseline symptom severity and psychosocial comorbidities. Notably, however, our final target of 36 patients is considerably larger than the minimum required sample size of 24 derived from these pilot estimates. This conservative oversampling strategy enhances the robustness of the study against data overdispersion and potential outliers, and ensures adequate statistical power for detecting the hypothesized treatment effect even with a moderate effect size. For a paired-sample *t*-test (α = 0.05, two-sided; power = 80%), 24 patients are required. To align with the sample size of the case–control component and to account for potential attrition, the final target for the patient group is set at 36 participants.

Thus, a total of 72 people will be included in the study sample: 36 patients with chronic tinnitus and 36 healthy controls who are matched for age and sex. This sample size ensures sufficient statistical power for both the between-group comparisons at baseline and the within-patient longitudinal analysis of treatment effects.

It should be noted that, although the primary longitudinal analysis will employ a two-way mixed-design analysis of variance (ANOVA) to evaluate the group × time interaction effect, the sample size calculations were conservatively based on simpler t-test frameworks (paired t-test for the matched baseline comparison, and paired t-test for the within-patient pre-post comparison). This approach is methodologically standard in clinical trial planning for the following reasons. First, sample size estimation is conventionally anchored to the most clinically meaningful single contrast to ensure adequate power for detecting the minimal effect of interest, without requiring assumptions about the covariance structure among repeated measurements. Second, mixed-design ANOVA generally provides equal or greater statistical power than pairwise *t*-tests for detecting time-related changes, as it accounts for the correlation structure among repeated measurements and pools variance more efficiently. Third, with 36 participants per group, the study is adequately powered not only for the primary t-test-based comparisons but also for the mixed ANOVA interaction test; post-hoc power calculations based on a moderate effect size (*f* = 0.25) for the group × time interaction indicates that 36 participants per group provide >85% power at α = 0.05 with two groups and two time points. Therefore, the sample sizes derived from the t-test assumptions represent a conservative lower bound that guarantees sufficient power even under the simplest analytical framework, while the subsequent application of mixed-design ANOVA will further enhance the sensitivity to detect the group × time interaction effect without compromising the validity of the study.

### 2.10 Evaluation of safety

The frequency of adverse events will be the main focus of the safety assessment. Any negative experiences, such as pain, bleeding, bruises, infection, and dizziness, will be reported by participants. At every evaluation, the researcher will record and assess all adverse occurrences on a standardized adverse event form, regardless of whether they are thought to be connected to the acupuncture therapy. Records will be kept of the onset time, duration, severity (mild, moderate, or severe), management strategy, resolution time, and causation assessment (definite, probable, improbable, or irrelevant).

## 3 Discussion

This study protocol is a two-phase clinical inquiry intended to assess the neurophysiological effects of acupuncture and investigate the connection between auditory brainstem function and chronic tinnitus. To our knowledge, this is one of the first studies to integrate ABR measurements with a standardized acupuncture intervention within a matched case–control and self-controlled design. This study is expected to provide insights into both the pathophysiological mechanisms of chronic tinnitus and the neurophysiological effects of acupuncture.

The first part of this study will compare the ABR parameters between patients and the healthy participants with age and sex matching. With the accumulation of existing evidence, we put forward a hypothesis that patients with chronic tinnitus will have specific ABR abnormalities, especially the amplitude reduction of wave I and (or) the prolongation of the latency of wave V. This hypothesis is based on the currently accepted model of cochlear synapsis, which refers to the selective loss of synapses between the internal hair cells and the auditory nerve. Even if the hearing threshold is normal, this kind of synaptic loss may occur (41). This condition, in which cochlear synapse loss occurs without affecting pure-tone thresholds, has been termed ‘hidden hearing loss’ (HHL) (22). In animal models, noise exposure can cause permanent loss of cochlear nerve synapses, with a loss rate of up to 50%. Although the cochlea threshold has been fully recovered at this time, the ABR wave I amplitude can still be used as a reliable non-invasive correlation indicator of synaptic density (42, 43). It has always been challenging to extrapolate these animal research findings to humans, but there are more and more successful cases. Bramhall et al. (2019) provided a comprehensive review of the methodological challenges and research progress faced in finding cochlear synaptic lesions caused by noise in humans. The conclusion of the review is that although the ABR wave I amplitude is still the most promising non-invasive biomarker, its application is limited by several factors, including height differences between subjects, individual differences in skull thickness, and limited dynamic range of the surface electrode itself (44). In order to overcome these limitations, many research teams have developed advanced signal processing technologies. Möhrle et al. (2019) adopted a new ABR experimental paradigm, which evaluates the defects in time processing through different click rates. Their research results showed that mice with evidence of behavioral tinnitus showed enhanced central nerve gain. This gain is considered to be a compensation mechanism for the loss of peripheral input. Through high-frequency stimulation, the above-mentioned neural gain can be observed (45).

The relationship between ABR abnormality and tinnitus perception will become more complicated due to individual differences in the central gain mechanism. Hofmeier et al. (46) conducted a large-scale study to find functional biomarkers that can distinguish between patients with and without hyperacusis. They found that patients with tinnitus without hyperacusis showed a prolonged latency and reduced amplitude of V waves in ABR. In contrast, patients with hearing sensitivity showed increased amplitude of III waves and V waves under high-intensity stimulation. This shows that the same peripheral damage may lead to differences in central compensation patterns due to different individual neurobiological factors. In the study of auditory brainstem response (ABR), methodological considerations are very important. A comprehensive systematic evaluation of the ABR scheme in tinnitus research by Milloy et al. (20) emphasized the importance of controlling participant characteristics, stimulation parameters, electrode placement and data analysis techniques, and pointed out that the significant heterogeneity between studies led to inconsistency in the results in the literature. In order to solve the above problems, this study adopts standardized click stimulation and tests under multiple stimulation intensities. In-ear headphones are used to optimize the attenuation effect of both ears. In addition, the study also implemented a strict quality control procedure: two independent audiologists who did not know about the group allocation interpreted the repeated recorded waveforms, respectively.

The second part of this study will evaluate the changes in ABR parameters after completing 10 acupuncture courses. In East Asia, acupuncture is widely used to treat tinnitus. Nevertheless, there is no sufficient evidence to decide whether to recommend acupuncture for the management of tinnitus. It shows that there is still an urgent need for rigorous clinical trials in this field (47). The quality of existing evidence is limited by several factors, including the small sample size, the lack of sham acupuncture controls, and the heterogeneity of the result measurement methods. These shortcomings are the goals that this study focuses on when designing. At the same time, emerging preclinical evidence shows that acupuncture may have a regulating effect on auditory function at multiple levels of the auditory pathway (48, 49). While these animal studies provide valuable mechanistic insights, their direct translation to the clinical setting requires careful consideration. Several factors limit the generalizability of these findings to our human protocol. First, animal models of tinnitus often use acute or trauma-induced conditions, whereas chronic tinnitus in humans typically evolves over years with complex peripheral and central interactions. Second, the electrophysiological end points differ: animal studies often measure ABR threshold shifts as indicators of hearing recovery, whereas our study focuses on supra-threshold latency and amplitude parameters as indicators of neural conduction and central gain. Despite these differences, the animal evidence supports the plausibility that acupuncture can modulate auditory brainstem activity, thereby providing a neurobiological rationale for our investigation. The present study will test whether these preclinical observations translate to measurable electrophysiological changes in human tinnitus patients. If the hypothesis of this study is confirmed, the findings would carry significant clinical and mechanistic implications. Demonstrating characteristic ABR profiles in chronic tinnitus patients would support the practical application of ABR as an objective biomarker for tinnitus subtyping, addressing a long-standing need for quantitative diagnostic tools in tinnitus research. Moreover, observing treatment-related changes in ABR parameters that correlate with clinical symptom improvement would provide neurophysiological evidence for acupuncture’s therapeutic effects, evidence that could extend beyond the current reliance on patient self-report and strengthen the rationale for future large-scale sham-controlled trials. The VA/DOD guidelines released in 2024 specifically put forward the following suggestions: verified subjective outcome measurement tools (such as tinnitus function index and tinnitus disorder list) should be used to monitor the treatment effect; at the same time, it is not recommended to use psychoacoustic measurement methods to achieve this purpose (47). However, the guide does not deal with electrophysiological measurement indicators such as auditory brainstem response (ABR). This omission highlights an important research gap, and this study may help fill this gap. Moreover, if treatment-related ABR parameter changes are observed, and these changes correspond to the improvement of clinical symptoms, then this will provide neurophysiological evidences for the therapeutic effect of acupuncture. These evidences can go beyond the current limitation of relying only on the subjective report of patients. This idea also coincides with the recent call for the use of objective outcome indicators in tinnitus clinical trials (50).

### 3.1 Limitations and future directions

This study has a number of limitations. Its sample size is comparatively modest because it is a single-center study. Therefore, the results may not be fully extended to a wider range of people with tinnitus. Another limitation is that the intervention group did not include a tinnitus control group receiving sham treatment, making it impossible to compare the effects of true acupuncture and sham acupuncture on patients with tinnitus. This is particularly worthy of attention, because the 2024 VA/DOD guidelines found insufficient evidence of acupuncture, and thus emphasized the need to carry out good controlled trials. While the self-controlled design allows each patient to serve as their own control and reduces between-subject variability, it cannot fully distinguish specific acupuncture effects from placebo effects, regression to the mean, or non-specific treatment effects. Although the inclusion of a healthy control group with repeated ABR measurements helps quantify normal test–retest variability, it does not replace a parallel sham-controlled arm. Therefore, our findings should be interpreted as preliminary and hypothesis-generating. Future adequately powered, sham-controlled randomized trials are needed to definitively establish the efficacy of acupuncture for chronic tinnitus. Third, the ABR protocol in this study uses broadband click stimuli rather than frequency-specific tone bursts. While this choice ensures clinical standardization and comparability with previous literature, it may reduce the sensitivity to detect frequency-specific cochlear synaptopathic changes that have been suggested to underlie some forms of tinnitus (51). Future studies incorporating multiple frequency-specific stimuli (e.g., 1, 2, and 4 kHz tone bursts) would provide a more comprehensive assessment of the relationship between acupuncture, ABR parameters, and tinnitus pathophysiology. Fourth, this study employs an assessor-blinded design rather than a double-blind design. Due to the nature of the acupuncture intervention, neither patients nor acupuncturists can be blinded to treatment allocation, which may introduce performance bias and expectation effects. To mitigate this, we have implemented several measures which have been motioned in the Design overview section. Nonetheless, we acknowledge that the lack of patient blinding remains a limitation. Fifth, treatment adherence is currently assessed primarily by session attendance, which does not fully capture treatment fidelity. To strengthen this aspect, all acupuncturists will follow a detailed Standard Operating Procedure (SOP) manual, needle manipulation parameters (angle, frequency, and duration) will be recorded for each session, and the De Qi sensation will be assessed using the C-MASS scale after each session. These additional measures will allow us to characterize the consistency of treatment delivery beyond simple attendance records. In addition, although ABR measurement is valuable for evaluating brainstem function, it cannot capture changes in the cortical level, which may also affect the perception of tinnitus. Lastly, this study did not set up a long-term follow-up beyond the treatment period, so it is impossible to evaluate whether the observed effect is persistent. And the 8-week follow-up assessment was conducted by telephone, which may introduce recall bias and reduce the accuracy of symptom evaluation compared with face-to-face assessment.

In order to overcome the above limitations, future research should adopt a multi-center experimental design, incorporate a larger sample size, set up a sham acupuncture control group, and extend the follow-up observation period. In addition, the inclusion of multimodal neuroimaging evaluation (such as resting functional MRI) in the research design will help to examine the mechanism of the brainstem and cortex at the same time. This practice may build a bridge between ABR’s research findings and network-level models. Furthermore, tinnitus can be classified into various subgroups based on clinical and electrophysiological traits. Therefore, future studies should consider adopting a larger sample size so that subgroup analysis can be carried out. The integration of genetics, psychology and neuroimaging measurement methods may eventually promote personalized treatment for this heterogeneous disease. This direction is also in line with the idea put forward by the recent theoretical frameworks (52).
